# Supplementary material for: IP3R-Mediated Calcium Release Promotes Ferroptotic Death in SH-SY5Y Neuroblastoma Cells
Source: Antioxidants (Basel). 2024 Feb 4;13(2):196. doi: 10.3390/antiox13020196 (PMC10886377; doi:10.3390/antiox13020196)
Supplement: Supplementary file 1 [file antioxidants-13-00196-s001.zip › antioxidants-2846219-supplementary.pdf]

Supplemental Figure S1.

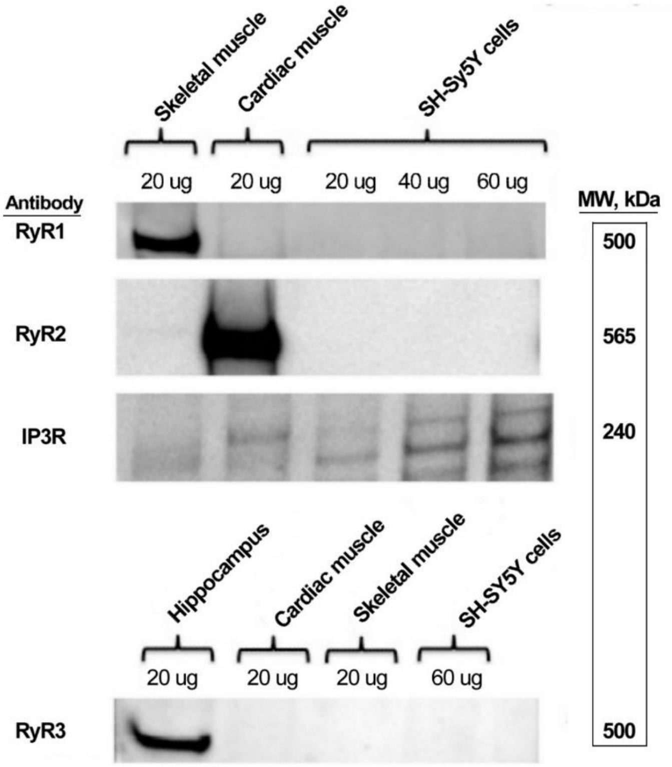

Supplemental Figure S1. Western blot analysis of RyR channel expression in SH-SY5Y cells. Representative Western blots of RyR1, RyR2, RyR3 and IP3R assessed in SH-SY5Y cells and positive controls tissues: skeletal muscle for RyR1 expression; cardiac muscle for RyR2 expression and hippocampus for RyR3 expression.

Supplemental Figure S2.

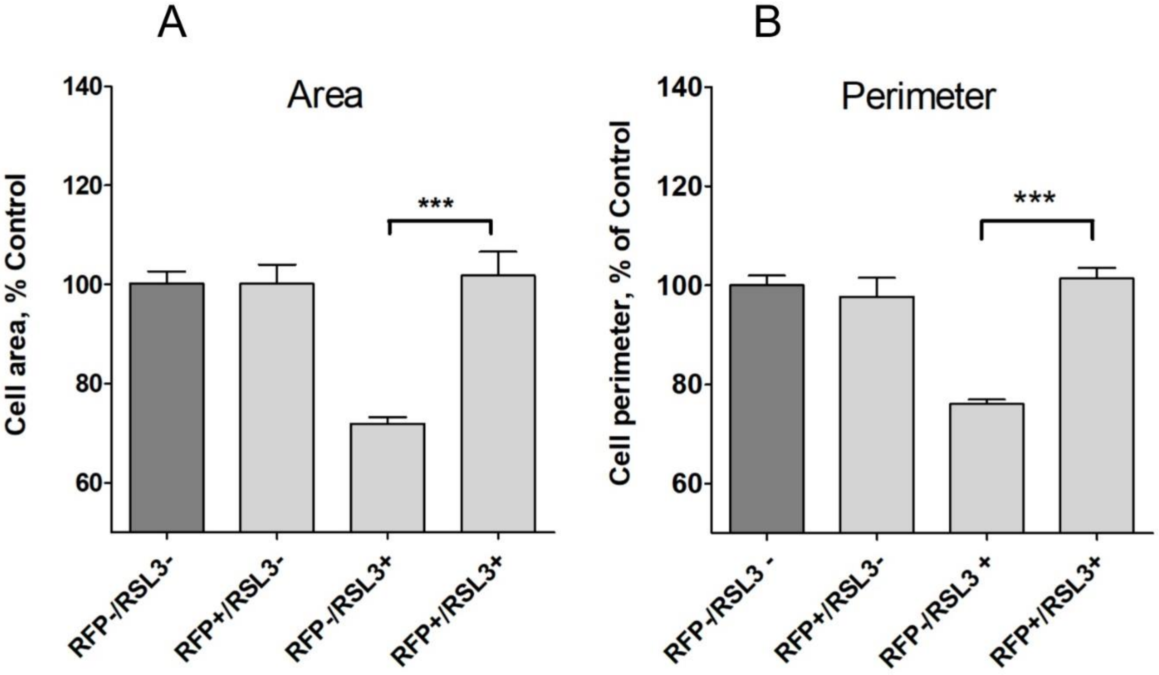

Supplemental Figure S2. Estimation of area (A) and perimeter (B) of RFP+ and RFP- cells treated or not with RSL3. Values represent mean ± SEM. Between 31 and 123 cells were evaluated per experimental condition from 2 independent experiments. ***P= 0.001 comparing the RFP-/RSL3+ and RFP+/RSL3+ conditions. No significant changes were detected between the RFP-/RSL3-, RFP+/RSL3- and RFP+/RSL3+ conditions.
